# Supplementary material for: Evaluation of the efficacy of sulbactam combination therapy for monomicrobial and polymicrobial pulmonary infections caused by multidrug-resistant Acinetobacter baumannii
Source: Microbiol Spectr. 2025 May 14;13(6):e03355-24. doi: 10.1128/spectrum.03355-24 (PMC12131869; doi:10.1128/spectrum.03355-24)
Supplement: Supplemental figures — Fig. S1 and S2. [file spectrum.03355-24-s0001.pdf]

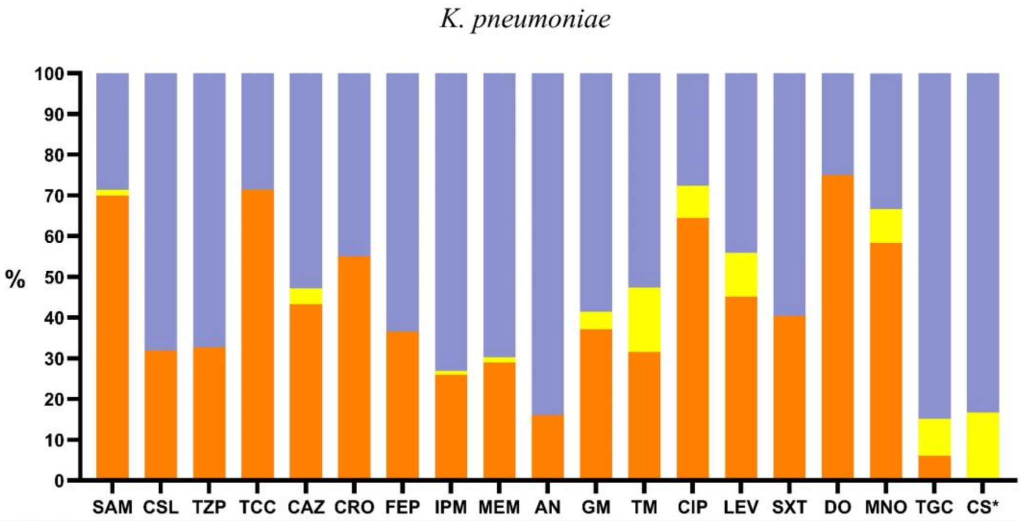

	SAM	CSL	TZP	TCC	CAZ	CRO	FEP	IPM	MEM	AN	GM	TM	CIP	LEV	SXT	DO	MNO	TGC	CS*
Resistant	70.00	31.91	32.69	71.43	43.27	55.10	36.54	25.96	28.95	16.00	37.14	31.58	64.47	45.10	40.38	75.00	58.33	6.06	0.00
Intermediate	1.43	0.00	0.00	0.00	3.85	0.00	0.00	0.96	1.31	0.00	4.29	15.79	7.89	10.78	0.00	0.00	8.33	9.09	16.67
Sensitive	28.57	68.09	67.31	28.57	52.88	44.90	63.46	73.08	69.74	84.00	58.57	52.63	27.63	44.12	59.62	25.00	33.33	84.85	83.33

Figure S1 Antimicrobial sensitivity patterns of *K. pneumoniae* clinical isolates against 19 antibiotics. Abbreviations (in the order in which they appear in the figure): SAM, ampicillin/sulbactam (2/1); CSL, cefoperazone/sulbactam (2/1); TZP, piperacillin/tazobactam; TCC, ticarcillin/clavulanic acid; CAZ, ceftazidime; CRO, ceftriaxone; FEP, cefepime; IPM, imipenem; MEM, meropenem; AN, amikacin; GM, gentamicin; TM, tobramycin; CIP, ciprofloxacin; LEV, levofloxacin; SXT, trimethoprim/sulfamethoxazole; DO, doxycycline; MNO, minocycline; TGC, tigecycline; CS, colistin. * Colistin and polymyxin B sensitivity results are equivalent.

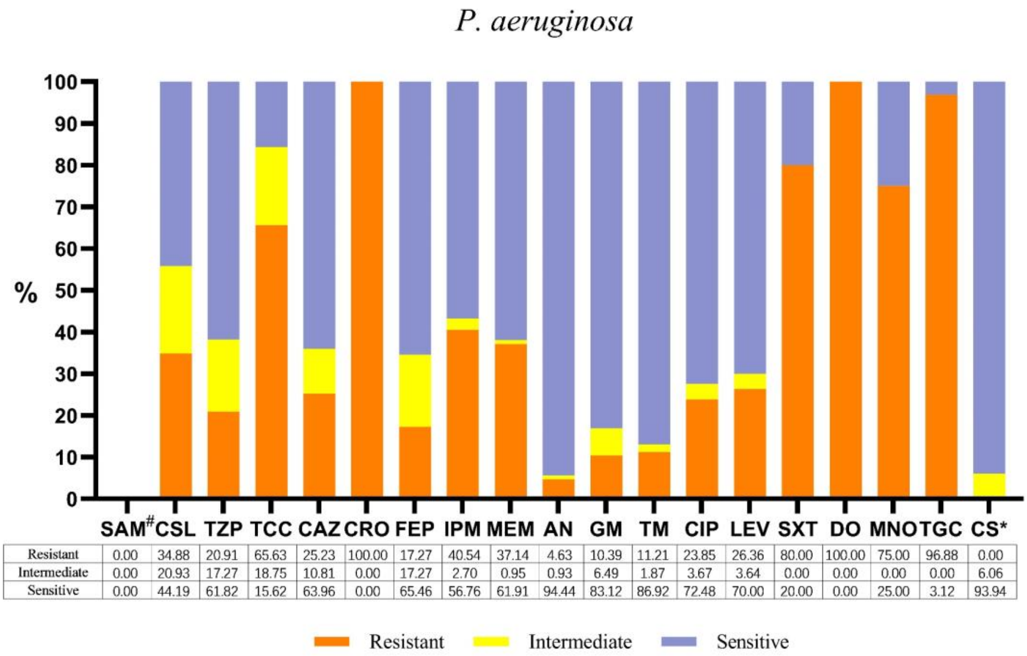

	SAM#	CSL	TZP	TCC	CAZ	CRO	FEP	IPM	MEM	AN	GM	TM	CIP	LEV	SXT	DO	MNO	TGC	CS*
Resistant	0.00	34.88	20.91	65.63	25.23	100.00	17.27	40.54	37.14	4.63	10.39	11.21	23.85	26.36	80.00	100.00	75.00	96.88	0.00
Intermediate	0.00	20.93	17.27	18.75	10.81	0.00	17.27	2.70	0.95	0.93	6.49	1.87	3.67	3.64	0.00	0.00	0.00	0.00	6.06
Sensitive	0.00	44.19	61.82	15.62	63.96	0.00	65.46	56.76	61.91	94.44	83.12	86.92	72.48	70.00	20.00	0.00	25.00	3.12	93.94

Figure S2 Antimicrobial sensitivity patterns of *P. aeruginosa* clinical isolates against 19 antibiotics. Abbreviations (in the order in which they appear in the figure): SAM, ampicillin/sulbactam (2/1); CSL, cefoperazone/sulbactam (2/1); TZP, piperacillin/tazobactam; TCC, ticarcillin/clavulanic acid; CAZ, ceftazidime; CRO, ceftriaxone; FEP, cefepime; IPM, imipenem; MEM, meropenem; AN, amikacin; GM, gentamicin; TM, tobramycin; CIP, ciprofloxacin; LEV, levofloxacin; SXT, trimethoprim/sulfamethoxazole; DO, doxycycline; MNO, minocycline; TGC, tigecycline; CS, colistin. # The drug sensitivity to *P. aeruginosa* was not tested. * Colistin and polymyxin B sensitivity results are equivalent.
