# Supplementary material for: Evaluation of the efficacy of sulbactam combination therapy for monomicrobial and polymicrobial pulmonary infections caused by multidrug-resistant Acinetobacter baumannii
Source: Microbiol Spectr. 2025 May 14;13(6):e03355-24. doi: 10.1128/spectrum.03355-24 (PMC12131869; doi:10.1128/spectrum.03355-24)
Supplement: Supplemental tables — Tables S1 to S3. [file spectrum.03355-24-s0002.pdf]

TABLE S1 Characteristics of 7-day microbial efficacy in study patients

**Characteristic**	**All patients (n=366)**	**Effective (n=235)**	**Invalid (n=131)**	***p*** [a]
Age (year, median [IQR])	67[55, 76]	67[54, 75]	68[56, 78]	0.282
Male	243 (66.4)	150 (63.8)	93 (71.0)	0.169
APACHE II score (median [IQR])	19[14, 22]	19[15, 22]	18[14, 22]	0.173
Mechanical Ventilation	295 (80.6)	197 (83.8)	98 (74.8)	**0.040**
Admitted to ICU	346 (94.5)	225 (95.7)	121 (92.4)	0.229
Treatment course (day, median [IQR])	10[6, 15]	11[7, 16]	9[6, 13]	**0.006**
Chronic underlying diseases	264 (72.1)	168 (71.5)	96 (73.3)	0.718
Diabetes	76 (20.8)	48 (20.4)	28 (21.4)	0.893
Hypertension	181 (49.5)	111 (47.2)	70 (53.4)	0.276
Heart disease	97 (26.5)	72 (30.6)	25 (19.1)	**0.019**
Cerebrovascular disease	63(17.2)	34 (14.5)	29 (22.1)	0.082
Malignant tumor	29 (7.9)	22 (9.4)	7 (5.3)	0.226
Complicated with infection other than pneumonia	147 (40.2)	100 (42.6)	47 (35.9)	0.223
Bloodstream infection	61 (16.7)	47 (20.0)	14 (10.7)	**0.028**
Urinary tract infection	55 (15.0)	37 (15.7)	18 (13.7)	0.650
Intracranial infection	17 (4.6)	8 (3.4)	9 (6.9)	0.193
Skin and soft tissue infection	15 (4.1)	13 (5.5)	2 (1.5)	0.096
Combined with other pathogens	291 (79.5)	199 (84.7)	92 (70.2)	**0.001**
Klebsiella pneumoniae	105 (28.7)	74 (31.5)	31 (23.7)	0.119
Pseudomonas aeruginosa	111 (30.3)	81 (34.5)	30 (22.9)	**0.024**
Stenotrophomonas maltophilia	54 (14.8)	43 (18.3)	11 (8.4)	**0.013**
Staphylococcus species	29 (7.9)	18 (7.7)	11 (8.4)	0.841
Escherichia coli	17(4.6)	12 (5.1)	5 (3.8)	0.618

Data are presented as median (25th-75th percentiles) or N (%). Statistically significant *p* values are highlighted in bold. APACHE, Acute Physiology and Chronic Health Evaluation; ICU, intensive care unit.

[a] *p* Values were calculated by *t*-test, Mann-Whitney *U*-test, or chi-square test as appropriate.

TABLE S2 Characteristics of 14-day clinical efficacy in study patients

**Characteristic**	**All patients (n=281)**	**Effective (n=108)**	**Invalid (n=98)**	**Uncertained (n=75)**	***p*** [a]
Age (year, median [IQR])	67[56, 74]	64.5[54, 72]	72[61.75, 82]	66[54, 73]	**< 0.001**
Male	177(63.0)	67 (62.0)	64 (65.3)	46 (61.3)	0.837
APACHE II score (median [IQR])	19[14.5, 22]	17[12, 21.75]	22[17, 23]	19[16, 22]	**< 0.001**
Mechanical Ventilation	233(82.9)	92 (85.2)	82 (83.7)	59 (78.7)	0.499
Admitted to ICU	270(96.1)	105 (97.2)	93 (94.9)	72 (96.0)	0.691
Treatment course (day, median [IQR])	12[9, 17]	12[9, 17]	12[9, 17]	12[9, 16]	0.664
Chronic underlying diseases	206(73.3)	77 (71.3)	81 (82.7)	48 (64.0)	**0.019**
Diabetes	63(22.4)	21 (19.4)	20 (20.4)	22 (29.3)	0.242
Hypertension	142(50.5)	51 (47.2)	56 (57.1)	35 (46.7)	0.268

Heart disease	76(27.0)	34 (31.5)	32 (32.7)	10 (13.3)	**0.007**
Cerebrovascular disease	38(13.5)	13 (12.0)	11 (11.2)	14 (18.7)	0.310
Malignant tumor	25(8.9)	9 (8.3)	10 (10.2)	6 (8.0)	0.851
Complicated with infection other than pneumonia	121(43.1)	47 (43.5)	42 (42.9)	32 (42.7)	0.992
Bloodstream infection	57(20.3)	19 (17.6)	23 (23.5)	15 (20.0)	0.576
Urinary tract infection	43(15.3)	17 (15.7)	10 (10.2)	16 (21.3)	0.130
Intracranial infection	8(2.8)	2 (1.9)	2 (2.0)	4 (5.3)	0.318
Skin and soft tissue infection	15(5.3)	4 (3.7)	7 (7.1)	4 (5.3)	0.548
Combined with other pathogens	233(82.9)	90 (83.3)	78 (79.6)	65 (86.7)	0.467
Klebsiella pneumoniae	91(32.4)	40 (37.0)	29 (29.6)	22 (29.3)	0.420
Pseudomonas aeruginosa	102(36.3)	37 (34.3)	39 (39.8)	26 (34.7)	0.671
Stenotrophomonas maltophilia	55(19.6)	24 (22.2)	18 (18.4)	13 (17.3)	0.667
Staphylococcus species	48(17.1)	20 (18.5)	14 (14.3)	14 (18.7)	0.660
Escherichia coli	18(6.4)	8 (7.4)	8 (8.2)	2 (2.7)	0.296

Data are presented as median (25th-75th percentiles) or N (%). Statistically significant *p* values are highlighted in bold.

[a] *p* Values were calculated by *t*-test, Mann-Whitney *U*-test, oneway analysis of variance (ANOVA), Kruskal-Wallis H(K) test, or chi-square test as appropriate.

TABLE S3 Characteristics of patients with sulbactam treatment ≥3-<7d and ≥7d in the 14-day clinical efficacy analysis

**Characteristic**	**Sulbactam treatment ⩾3-<7 days (n=85)**	**Sulbactam treatment ⩾7 days (n=281)**	***p*** [a]
Age (year, median [IQR])	69[55, 79.5]	67[56, 74]	0.150
Male	68(80.0)	177(63.0)	**0.004**
APACHE II score (median [IQR])	19[14, 23]	19[14.5, 22]	0.998
Mechanical Ventilation	62(72.9)	233(82.9)	**0.044**
Admit to ICU	76(89.4)	270(96.1)	**0.036**
Treatment course (day, median [IQR])	5[4, 6]	12[9, 17]	＜**0.001**
**Chronic underlying diseases**	59(69.4)	206(73.3)	0.491
Diabetes	18(21.2)	63(22.4)	0.882
Hypertension	41(48.2)	142(50.5)	0.805
Heart disease	21(24.7)	76(27.0)	0.678
Cerebrovascular disease	23(27.1)	38(13.5)	**0.005**
Malignant tumor	3(3.5)	25(8.9)	0.110
**Complicated with infection other than pneumonia**	31(36.5)	121(43.1)	0.316
Bloodstream infection	10(11.8)	57(20.3)	0.080
Urinary tract infection	9(10.6)	43(15.3)	0.296
Intracranial infection	7(8.2)	8(2.8)	0.060
Skin and soft tissue infection	2(2.4)	15(5.3)	0.394
**Combined with other pathogens**	64(75.3)	233(82.9)	0.153
Klebsiella pneumoniae	25(29.4)	90(32.0)	0.691
Pseudomonas aeruginosa	21(24.7)	101(35.9)	0.066
Stenotrophomonas maltophilia	10(11.8)	53(18.9)	0.143
Staphylococcus species	6(7.1)	46(16.4)	**0.033**
Escherichia coli	3(3.5)	17(6.0)	0.533
**Sulbactam daily dosage**			
⩽4g (ref)	69(81.2)	192(68.3)	0.069
≥6g- < 8g	9(10.6)	46(16.4)	
≥8g	7(8.2)	43(15.3)	
**Sulbactam combination regimens**			
Sulbactam-based (ref)	43(50.6)	92(32.7)	**0.012**
Sulbactam + tigecycline	28(32.9)	109(38.8)	
Sulbactam + polymyxin	8(9.4)	32(11.4)	
Sulbactam + polymyxin + tigecycline	6(7.1)	48(17.1)	

Data are presented as median (25th-75th percentiles) or N (%). Statistically significant p values are highlighted in bold. APACHE, Acute Physiology and Chronic Health Evaluation; ICU, intensive care unit.

[a] *p* Values were calculated by ManneWhitney *U*-test, or chi-square test as appropriate.
